# Significance of Correlation of Shear Wave Elastography With Fibrosis-4 in a Cohort of Patients With Diabetes and Nonalcoholic Fatty Liver Disease

**DOI:** 10.7759/cureus.67015

**Published:** 2024-08-16

**Authors:** Rohan Kelkar, Uday Phadke, Raveena Kelkar, Shalmi Khanapurkar, Nishad A Barve

**Affiliations:** 1 Internal Medicine, Sahyadri Super Speciality Hospital, Deccan Gymkhana, Pune, IND; 2 Endocrinology, Diabetes and Metabolism, Sahyadri Super Speciality Hospital, Deccan Gymkhana, Pune, IND; 3 Internal Medicine, Cleveland Clinic Akron General, Akron, USA; 4 Internal Medicine, Deenanath Mangeshkar Hospital, Pune, IND; 5 Internal Medicine, Cleveland Clinic Mercy, Canton, USA

**Keywords:** fibrosis, liver cirrhosis, liver elastography, fib-4, diabetes mellitus type 2, nonalcoholic fatty liver disease (nafld)

## Abstract

Background

Nonalcoholic fatty liver disease (NAFLD) is increasingly recognized as a cause of chronic liver disease. It can lead to complications such as decompensated liver cirrhosis and hepatocellular carcinoma.

Objectives

This study aimed to assess liver stiffness using point shear wave elastography in patients with diabetes and NAFLD and to compare the results with the FIB-4 (fibrosis-4) score, AST/ALT (aspartate aminotransferase-to-alanine aminotransferase) ratio, and APRI (AST-to-Platelet Ratio Index).

Materials and methods

A cross-sectional study was conducted on type 2 diabetes patients who underwent point shear wave liver elastography for liver stiffness estimation between January 2020 and February 2023. Demographic data such as age, sex, and laboratory data (AST, ALT, and platelet count) were recorded. FIB-4 score, APRI, and AST/ALT ratio were calculated for these patients. The results of the FIB-4 score and APRI were then compared with the shear wave liver elastography fibrosis scores.

Results

The analysis included 60 patients, of whom 50 (83.33%) were male, with a mean age of 44.8 years (SD: 11.02; range: 21-69). Thirty-six patients (60%) had significant fibrosis. There was a significant positive correlation between the shear wave elastography results and the FIB-4 and APRI scores.

Conclusion

The findings revealed that nearly two-thirds of the study group had significant fibrosis (≥F2), highlighting the need for early NAFLD diagnosis and treatment. Noninvasive laboratory serum markers, in conjunction with shear wave liver elastography, are useful for diagnosing severe fibrosis.

## Introduction

Nonalcoholic fatty liver disease (NAFLD) involves excessive buildup of fat in the liver (known as steatosis) that is not attributable to heavy alcohol consumption or other secondary factors. These secondary factors may include medication use, hepatitis C infection, and various endocrine disorders. NAFLD includes a range of conditions, spanning from simple steatosis to the more severe inflammatory state known as nonalcoholic steatohepatitis (NASH), which can develop into fibrosis and cirrhosis [1]. Some NAFLD patients may experience liver failure, develop hepatocellular carcinoma (HCC), or require a liver transplant. NAFLD is often associated with conditions like type 2 diabetes and metabolic syndrome and is regarded as their liver manifestation [2]. The prevalence of NAFLD in type 2 diabetes mellitus (T2DM) patients is 59.67% [3]. Research suggests that over 30% of NAFLD patients may progress to NASH, while approximately 25% develop fibrosis, 10%-20% advance to cirrhosis, and around 4% may even develop HCC [4,5]. Consequently, accurately diagnosing disease progression becomes crucial for determining treatment strategies and prognosis, as they rely heavily on the severity of liver fibrosis histologically. While liver biopsy is commonly regarded as the "gold standard" for diagnosing NAFLD [6], its invasive nature, potential complications, and cost limit its utility. Point shear wave elastography uses ultrasound waves for the noninvasive staging of fibrosis in patients with NAFLD [7]. Minimally invasive laboratory markers for liver fibrosis assessment include the aspartate aminotransferase/alanine aminotransferase (AST/ALT) ratio, fibrosis-4 (FIB-4) score, and AST to platelet ratio index (APRI) [8].

Objectives

This study compared liver fibrosis assessment results obtained through point shear wave elastography with FIB-4, APRI, and AST/ALT measurements in type 2 diabetes patients diagnosed with NAFLD during the time period from January 2020 to February 2023.

## Materials and methods

The research adhered to the guidelines outlined in the Declaration of Helsinki and received approval from the hospital's Biomedical and Health Research Committee (BHRC). This was a prospective study conducted from March 2022 to February 2023. Retrospective data of patients were collected from January 2020 through February 2022. Patients who presented to the Diabetes and Endocrinology Outpatient Department of Sahyadri Hospital, Deccan Gymkhana, Pune, India, were selected.

To achieve a power of 90% and a significance level of 5%, using a correlation coefficient of 0.420 as a reference, the sample size was calculated according to a formula outlined in the study by Hulley et al. [9]. The calculation determined the total sample size (N) using the equation [(Z α + Z β) / C]² + 3, where C equals 0.5 times the natural logarithm of [(1 + r) / (1 - r)]. With the given values, including r as 0.420, Z α as 1.9600, Z β as 1.2816, and C as 0.4477, the formula yielded a total sample size of approximately 55 individuals.

Type 2 diabetes patients diagnosed with NAFLD based on point shear wave liver elastography performed at our hospital were selected, and FIB-4 and APRI measurements were calculated. Patients with complete blood test results who had undergone point shear wave elastography were included, while those with incomplete data were excluded.

Exclusion criteria included patients with chronic hepatitis B or C, autoimmune hepatitis, or alcoholic liver disease. Additionally, individuals with advanced liver or cardiac failure, as well as those exhibiting signs of decompensated cirrhosis, were also excluded from the study. The standard laboratory reference range for serum ALT is 1-45 U/L, and for AST, it is 1-40 U/L. The standard reference range for platelet count is 150-410 k/μL. The FIB-4 Index is a minimally invasive tool developed to estimate the presence of advanced liver fibrosis. It was calculated using the following formula [10]: FIB-4 = Age (years) × AST (U/L) / [PLT (10⁹/L) × ALT 1/2 (U/L)]. The APRI is a tool for assessing liver fibrosis, calculated using the equation [11]: APRI = [(AST / upper limit of the normal AST range) × 100] / Platelet Count.

According to previous reports and meta-analyses, significant fibrosis was defined as FIB-4 values ≥1.3 [12] and APRI values ≥0.5 [13].

Referring to the consensus statement for the diagnosis of obesity, abdominal obesity, and metabolic syndrome in Asian Indians [14], the patients’ body mass index (BMI) and waist circumference were analyzed (Table 1).

**Table 1:** Consensus statement for the diagnosis of obesity, abdominal obesity, and metabolic syndrome for Asian Indians.

Category	Criteria	Classification
BMI (kg/m²)	18.0-22.9	Normal
	23.0-24.9	Overweight
	>25	Obesity
Abdominal obesity (cm)		
Males	>90	Abdominal obesity
Females	>80	Abdominal obesity

Point shear wave elastography (PSWE) is an ultrasound-based technique that uses a focused ultrasound beam to generate shear waves in the liver. It estimates liver stiffness by measuring and monitoring the velocity and propagation of these shear waves. As fibrosis progresses, liver tissue becomes stiffer, resulting in faster wave propagation. By measuring the wave propagation velocity, the degree of stiffness, and consequently the stage of liver fibrosis, can be determined.

PSWE was performed using the ElastPQ technique for fibrosis estimation with the Philips Affiniti 70 ultrasound machine (Philips Healthcare, Bothell, Washington), which has an inbuilt point shear wave elastography software. At least 10 measurements were taken in segments 7 and 8 of the liver during a brief breath hold (of a few seconds). The results were presented in kilopascals, with the median value considered representative. Quality parameters like IQR/M were used to optimize performance [15,16].

The Meta-analysis of Histological Data in Viral Hepatitis (METAVIR) is a scoring system used to assess liver fibrosis in a liver biopsy sample. Patients were grouped into different METAVIR fibrosis stages based on their PSWE results, using the specified cutoff values provided by Philips. Specifically, individuals with PSWE readings ranging from 2.0 to 4.5 kPa were classified as normal (F0); those with readings between 4.5 and 5.7 kPa were designated as fibrosis stages F0-F1; patients with readings between 5.7 and 12.0 kPa were categorized as stages F2-F3; and those with PSWE values between 12 and 21 kPa were allocated to stages F3-F4.

Statistical analysis

Data analysis was performed using IBM SPSS Statistics for Windows, Version 20 (Released 2011; IBM Corp., Armonk, New York). The categorical variables were expressed as percentages, while the quantitative variables were expressed as mean ± SD and median with IQR. Student’s t-test was employed to determine the differences in mean ages between male and female participants. Spearman's correlation analysis was used to evaluate the relationship between shear wave elastography results and age, platelet count, and serum ALT level. Additionally, Spearman's correlation analysis was conducted to assess the relationship between shear wave elastography results and AST/ALT ratio, FIB-4, and APRI scores. The Mann-Whitney U test was utilized to evaluate differences in FIB-4, APRI, and AST/ALT measurements between patients with significant fibrosis (≥F2) and those with mild or no fibrosis (F0-F1 or F0).

## Results

The final analysis comprised 60 patients, including 50 (83.3%) males and 10 (16.7%) females. The mean age was 44.8 years, with a standard deviation of 11.02 years, ranging from 21 to 69 years. Nineteen (31.7%) were in the age group of <40 years, 19 (31.7%) in the 40-49 years age group, 17 (28.3%) in the 50-59 years age group, and 5 (8.3%) were 60 years or older. The mean BMI was 28.59 ± 4.04 kg/m². One (1.6%) patient had a normal BMI, 10 (16.7%) were overweight, and 49 (81.7%) were obese. Additionally, 59 (98.3%) patients had abdominal obesity. Table 2 presents the baseline characteristics of the NAFLD patients.

**Table 2:** Demographic characteristics of NAFLD patients NAFLD: nonalcoholic fatty liver disease.

Variable	No.	%
Age, mean ± SD (44.8±11.02 years)		
<40 years	19	31.67
40-49 years	19	31.67
50-59 years	17	28.33
≥60 years	5	8.33
Gender		
Male	50	83.3
Female	10	16.67
BMI		
Normal	1	1.66
Overweight	10	16.66
Obese	49	81.66
Abdominal obesity (waist circumference)		
Males		
>90 cm	49	81.66
≤90 cm	1	1.66
Females		
>80 cm	10	16.66
≤80 cm	0	0
Total	60	100

The median stiffness score by elastography was 6.15 kPa (interquartile range: 2.23 kPa), and most patients exhibited significant fibrosis (≥F2). Table 3 shows the distribution of patients according to the stage of fibrosis.

**Table 3:** Stage of fibrosis

Stage	No.	%
F0 (2.0‐4.5 kPa)	4	6.67
F0-F1 (4.5‐5.7 kPa)	20	33.33%
F2-F3 (5.7‐12.0 kPa)	33	55%
F3-F4 (12-21 kPa)	3	5%
Total	60	100

Using the independent samples Mann-Whitney U test, no significant differences were noted in age, ALT, platelet count, BMI, and waist circumference between males and females. Additionally, there were no significant differences in FIB-4, APRI, AST/ALT, and PSWE measurements between males and females.

Spearman's correlation coefficient was determined to assess the correlation between shear wave elastography and variables such as age, BMI, waist circumference, ALT, and platelet count. No significant correlations were found. Additionally, there was no significant difference in ALT, BMI, and waist circumference between those with ≥F2 fibrosis and those with <F2 fibrosis.

**Figure 1:**
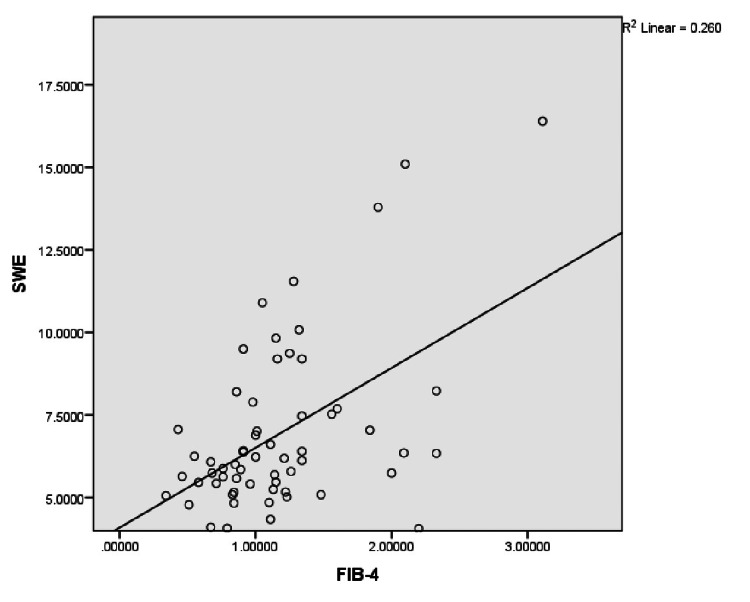
Coefficient of determination (R2) between PSWE and FIB-4 PSWE: point shear wave elastography, FIB-4: fibrosis-4.

However, Spearman’s correlation coefficient revealed a significant correlation (Figure 1) between FIB-4 and shear wave elastography measurements (r = 0.424, p = 0.001) as well as between APRI and shear wave elastography (r = 0.321, p = 0.012). The AST/ALT ratio did not significantly correlate with PSWE values (r = 0.19).

**Figure 2:**
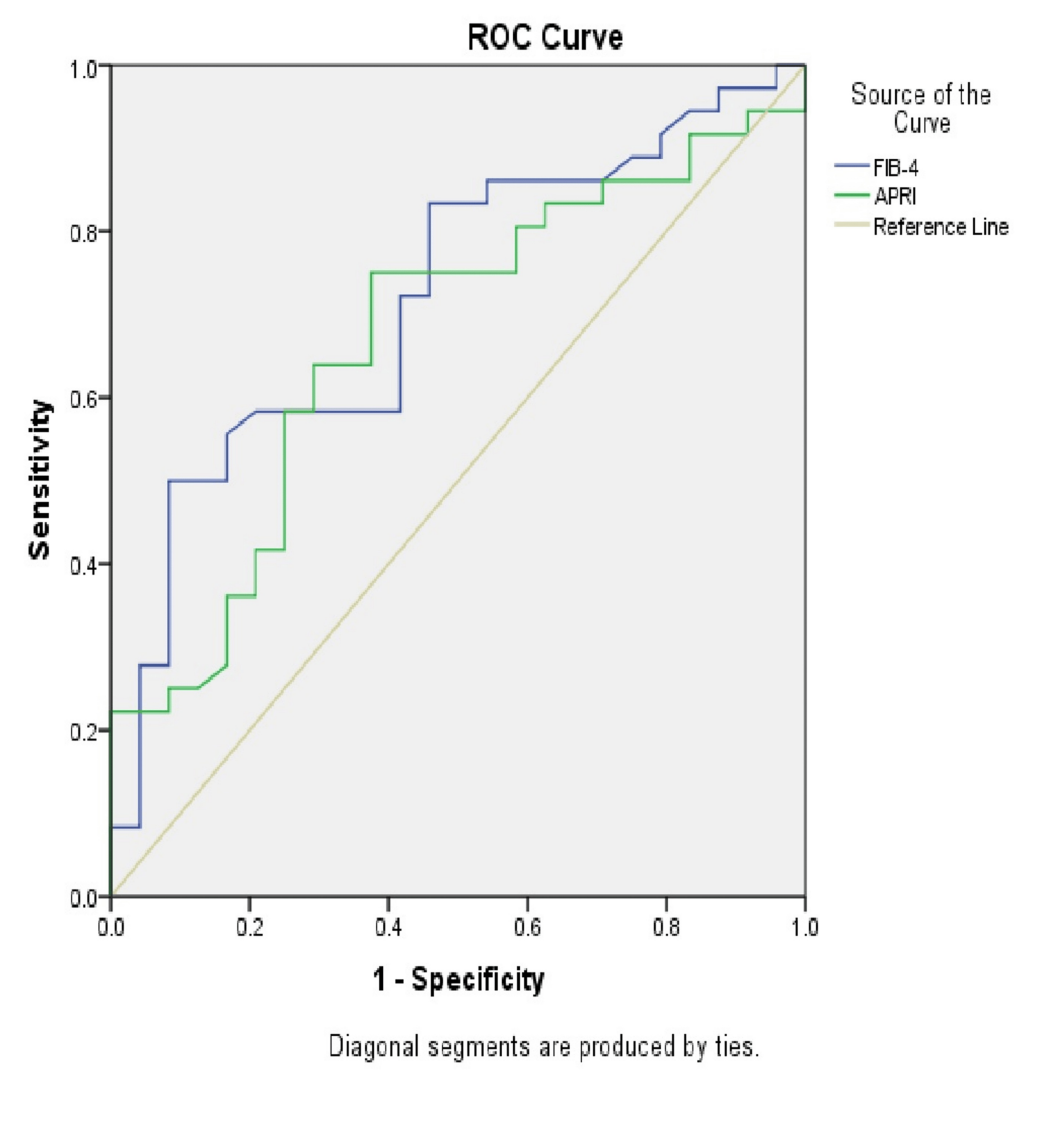
ROC curve for FIB-4 and APRI for prediction of significant fibrosis (≥F2) ROC: receiver operating characteristic, FIB-4: fibrosis-4, APRI: Aspartate Aminotransferase to Platelet Ratio Index.

Figure 2 demonstrates the receiver operating characteristic (ROC) curves for FIB-4 and APRI in predicting significant fibrosis. For FIB-4, the area under the ROC curve (AUROC) to predict ≥F2 was 0.719; a cutoff of 0.9 had a sensitivity of 80.6% and a specificity of 54.2%. For APRI, the AUROC to predict ≥F2 was 0.672; a cutoff of 0.4625 had a sensitivity of 75% and a specificity of 62.5%.

The chi-square test was used to assess the association between FIB-4 ≥1.3 and PSWE measurements corresponding to ≥F2, which was found to be significant (p = 0.005). Additionally, the chi-square test was used to evaluate the association between APRI ≥0.5 and PSWE measurements corresponding to ≥F2, which was also significant (p = 0.014) (Table 4).

**Table 4:** Association between FIB-4 and APRI groups and significant fibrosis (≥F2) p < 0.05 is considered significant. FIB-4: fibrosis-4, APRI: Aspartate Aminotransferase to Platelet Ratio Index.

Groups	< F2	≥F2	p-value
FIB-4 <1.3	22	21	0.005
FIB-4 ≥1.3	2	15	
Total	24	36	
APRI <0.5	15	11	0.014
APRI ≥0.5	9	25	
Total	24	36	

A significant difference was observed in the FIB-4 and APRI calculations between patients with significant fibrosis (≥F2) and those with mild or no fibrosis (<F2) using the nonparametric Mann-Whitney U-test (p = 0.004 and p = 0.025, respectively). No significant difference was observed in the distribution of the AST/ALT ratio between the two groups. 

## Discussion

Our data show a high percentage of males with NAFLD, consistent with previous studies from India [17,18]. The average age of NAFLD patients in our study was 44.8 years, aligning with findings from earlier studies conducted in the Indian population [19,20].

The mean BMI in our study was 28.59, which matches previous studies [21,22], and 98.33% of patients were overweight or obese (BMI >23) according to the Asia Pacific criteria for obesity. The higher prevalence of obesity may be attributed to using more stringent criteria compared to previous studies, as well as the fact that all patients had type 2 diabetes.

A significant correlation was observed between the FIB-4 score and PSWE measurement (r = 0.424; p = 0.001) and between APRI and PSWE measurement (r = 0.321; p = 0.012), which approximates the findings in other studies using transient elastography [23,24]. Additionally, a significant association was found between ≥F2 fibrosis and high FIB-4 (≥1.3) and APRI (≥0.5), highlighting the utility of FIB-4 and APRI indices in predicting significant fibrosis (≥F2).

The data indicated that a considerable proportion (60%) of the NAFLD patients demonstrated significant liver fibrosis (≥F2) as per the point shear wave elastography examinations, aligning with findings from a study conducted by Kuchay et al. [25]. These findings were reinforced by the significant correlation observed between the shear wave liver elastography results (Figure 1) and the FIB-4 and APRI scores.

A substantial correlation between PSWE and the AST/ALT ratio was not observed. In a study by Li et al. [26], the AST/ALT ratio in advanced NAFLD patients was normal, which coincides with the data from our study. The AST/ALT ratio showed the lowest diagnostic performance in differentiating ≥F2 from <F2.

Razavizade et al.'s research [27] emphasizes the significance of integrating serum markers with ultrasound evaluations for categorizing NAFLD patients based on disease severity. This combined approach offers a practical and cost-effective alternative, particularly in settings where advanced diagnostic methods like transient elastography or magnetic resonance elastography are unavailable, or where liver biopsy is not warranted. By simplifying the diagnostic process and potentially reducing the need for invasive procedures like liver biopsy, this strategy enhances patient care and facilitates informed treatment decisions. In essence, it highlights the importance of utilizing multiple diagnostic modalities to manage NAFLD patients effectively.

Therefore, utilizing PSWE examinations alongside FIB-4 and APRI scores offers a beneficial approach for monitoring early-stage NAFLD patients in situations where liver biopsy lacks clear indication. Moreover, individuals undergoing bariatric surgery or other NAFLD therapies can be appropriately monitored using these noninvasive diagnostic techniques.

Limitations

While the study does have some limitations, they do not significantly undermine the overall findings. The absence of liver biopsy, although considered the gold standard, is mitigated by the use of alternative noninvasive techniques such as PSWE. The small sample size, while not ideal, still provides valuable insights and trends that can guide future research. The limited number of published articles on PSWE in NAFLD patients highlights an area ripe for exploration rather than a fundamental flaw. Lastly, although the lack of standardized fibrosis scoring among ultrasound vendors could introduce some variability, this is a common challenge in medical imaging research and can be addressed through standardized protocols and calibration procedures.

## Conclusions

In summary, our research offers valuable insights into the demographic profiles and diagnostic efficacy of noninvasive techniques for evaluating NAFLD in India. Our findings highlight significant correlations between FIB-4 and APRI scores with PSWE measurements, suggesting the potential of these noninvasive tools in detecting liver fibrosis. The high prevalence of significant fibrosis among our NAFLD cohort underscores the importance of timely diagnosis and the integration of serum markers alongside ultrasound examinations offers promise in categorizing NAFLD severity when more advanced diagnostic techniques are unavailable. Overall, our study supports the use of FIB-4 and APRI indices as effective tools for the noninvasive assessment of NAFLD patients, particularly in settings where liver biopsy is not feasible or warranted. These findings contribute to advancing clinical practices for NAFLD management in India and beyond.
